# Interleukin-22: a potential therapeutic target in atherosclerosis

**DOI:** 10.1186/s10020-021-00353-9

**Published:** 2021-08-13

**Authors:** Jin-Wen Luo, Yuan Hu, Jian Liu, Huan Yang, Peng Huang

**Affiliations:** 1 Department of Cardio-Thoracic Surgery, Hunan Children’s Hospital, Changsha, 410007 People’s Republic of China; 2 Department of Ultrasound Medicine, Hunan Children’s Hospital, Changsha, 410007 People’s Republic of China; 3 Department of Respiratory Medicine, Hunan Provincial People’s Hospital, Changsha, Hunan 410001 People’s Republic of China

**Keywords:** Atherosclerosis, IL-22, Inflammatory response, Cytokine, Cholesterol metabolism

## Abstract

**Background:**

Atherosclerosis is recognized as a chronic immuno-inflammatory disease that is characterized by the accumulation of immune cells and lipids in the vascular wall. In this review, we focus on the latest advance regarding the regulation and signaling pathways of IL-22 and highlight its impacts on atherosclerosis.

**Main body:**

IL-22, an important member of the IL-10 family of cytokines, is released by cells of the adaptive and innate immune system and plays a key role in the development of inflammatory diseases. The binding of IL-22 to its receptor complex can trigger a diverse array of downstream signaling pathways, in particular the JAK/STAT, to induce the expression of chemokines and proinflammatory cytokines. Recently, numerous studies suggest that IL-22 is involved in the pathogenesis of atherosclerosis by regulation of VSMC proliferation and migration, angiogenesis, inflammatory response, hypertension, and cholesterol metabolism.

**Conclusion:**

IL-22 promotes the development of atherosclerosis by multiple mechanisms, which may be a promising therapeutic target in the pathogenesis of atherosclerosis.

## Introduction

Cardiovascular diseases (CVDs) represent the major factor of disability and premature death worldwide. Recently, the World Health Organization (WHO) revealed that CVD accounts for > 17 million deaths per year and this figure will rise to over 23 million by the year 2030 (GBD 2015 Risk Factors Collaborators 2016; Li et al. 2021a). It is well known that atherosclerosis is an inflammatory disease of the vascular wall, which is recognized as the underlying basis of many CVDs, such as hypertension, myocardial infarction, stroke, and peripheral arterial disease. The development and pathogenesis of atherogenesis are closely associated with many pathophysiological factors, such as inflammatory response, angiogenesis, hypertension, vascular smooth muscle cell (VSMC), and cholesterol metabolism.

Interleukin-22 (IL-22), discovered in 2000, is known as a crucial member of the IL-10 cytokine family (Dumoutier et al. 2000). Since the discovery of IL-22, its biological functions have been extensively studied. IL-22 can be produced by lymphoid lineage cells (Th1, Th17, Th22, γδT, etc.) and innate lymphocyte cells. Also, IL-22 was reported to have an important function in inflammation, cell proliferation, tissue repair, and host defense (Rutz et al. 2013; Mühl et al. 2013; Eidenschenk et al. 2014; Sonnenberg et al. 2011). Although Th1 and Th17 cells play a role in the development of atherosclerosis, Th22 cell-derived IL-22 is more important in atherosclerosis (Shi et al. 2020a). Several lines of evidence have demonstrated that IL-22 aggravates myocardial infarction, hypertension, cardiac hypertrophy, and myocarditis (Zhang et al. 2013; Ye et al. 2017; Kong et al. 2013; Kong et al. 2014). Recently, numerous studies suggested that IL-22 promotes atherosclerosis through multiple mechanisms including promotion of inflammation, regulation of cholesterol metabolism, and induction of VSMC proliferation and migration (Rattik et al. 2015; Chellan et al. 2014), indicating this cytokine as a promising and novel target to treat or prevent atherosclerotic cardiovascular disease. In the present review, we summarize the current knowledge about the regulation and signaling pathways of IL-22 and highlight its roles in atherogenesis.

## Structural features of IL-22

The IL-22 gene is located on human chromosome 12 at q15 and encodes a protein with a length of 179 amino acids. After enzymatic modification of its signal peptide (33 amino acids), the cytokine is secreted as a 146-amino-acid protein (Nagem et al. 2002; Bleicher et al. 2008). Human IL-22 shares 79% amino acid sequence identity with the mouse type. The precursor gene of IL-22 is approximately 5257 base-pair long and consists of 5 exons encoding IL-22, and then converted into a secreted form (Xu et al. 2005; Manna et al. 2017). The crystallographic structure of recombinant human IL‑22 (expressed in Drosophila melanogaster and Escherichia coli) was solved using X‑ray diffraction and crystallization (Nagem et al. 2002; Xu et al. 2005). The IL-22 monomer has a compact bundle of a single domain that is composed of six interconnected α‑helices (A to F) and connecting loops (Manna et al. 2017; Wei et al. 2019; Nagem et al. 2006). Its basic structure contains two intramolecular disulfide bonds and four cysteines.

IL-22 is biologically active as a monomer. However, it has also been reported that there are functional tetramer and dimer forms of IL-22 under high concentrations in solution (Nagem et al. 2002; Bleicher et al. 2008; Oliveira et al. 2008). The molecular weight of IL-22 is approximately 16.7 kDa, with three potential N‑linked glycosylation sites including loop AB (Asn68-Asn69-Thr70), helix A (Asn68-Arg55-Thr56), and helix C (Asn97-Phe98-Thr99). It is believed that glycosylation is only related to minor changes in the tertiary structure of IL-22. However, various genetic polymorphisms including insertion-deletion (indel), single nucleotide polymorphism, somatic single nucleotide variants, and nucleotide deletion, have been identified as the functional modulators of IL-22 gene (Shohan et al. 2020). When producing therapeutically usable neutralizing IL-22-specific monoclonal antibodies (MAbs), the glycosylation form of the gene should be considered.

## IL-22 receptors and signaling

The IL-22 receptor (IL-22R) is part of the family of class II cytokine receptors and is composed of two heterodimeric subunits, IL-10R2 (also named IL10RB) and IL-22R1 (Xie et al. 2000; Sabat et al. 2014; Li et al. 2004). The human genes for IL-10R2 and IL-22R1 proteins are located on chromosomes 21q22.11 and 1p36.11, respectively. Both receptors have three similar domains: transmembrane, extracellular, and an intracellular signaling region (Bleicher et al. 2008; Xie et al. 2000). Extracellular motifs of the IL-22R complex consists of N-linked glycosylation sites, two fibronectin type III domains, and conserved amino acids (Xie et al. 2000; Langer et al. 2004). Moreover, it has been reported that the longer intracellular moiety of IL-10R2 (79 amino acid sequence) and IL-22R1 (325 amino acid sequence) consist of four Tyr-x-x-Gln motifs, which are putative signal transducer and activator of transcription (STAT) recruitment sites (Kotenko et al. 1997; Kotenko et al. 2001). Of note, IL-22 protein has a strong binding affinity for IL-22R1 (K_D_ ranging from 1 ~ 20 nM) but no affinity for IL‑10R2 (Logsdon et al. 2002; Logsdon et al. 2004; Jones et al. 2008a). However, studies have observed that the IL-10R2 subunit has a high interaction affinity for the IL-22/IL-22R1 complex (Jones et al. 2008b). The binding of IL-22 to IL-22R1 would cause the conformational change of IL-22 protein, which promotes the secondary binding of the IL-22/IL22R1 complex to IL-10R (Bleicher et al. 2008; Li et al. 2004). Of note, a study by Weathington et al. found that the serine residue at positions 414 and 410 of the IL-22R1 gene sequence are phosphorylated under the enzymatic reaction of the kinase glycogen synthase kinase 3β (GSK3β), which promotes the stability of IL-22R and inhibits its degradation via the ubiquitin proteasome (Weathington et al. 2014).

Besides the cell surface-bound IL-22R complex, a soluble IL-22-binding receptor, known as IL-22 binding protein (IL-22BP) or L-22RA2, has been identified to bind IL-22 with high affinity. IL-22BP gene is located on human chromosome 6q23.3. This gene encodes a protein with 210 amino acid residues that share 34% sequence homology to the IL-22R1 subunit (Gruenberg et al. 2001; Dumoutier et al. 2001; Wei et al. 2003; Xu et al. 2001). It has been reported that IL-22BP interacts with IL-22 at the binding sites overlapping with IL-22R1, which directly blocks the binding of IL‑22 to its receptor complex (Wu et al. 2008). In addition, the binding affinity of IL-22 to IL-22BP is up to1000-fold higher than that between IL‑22R1 and IL‑22 (Wolk et al. 2007; Dudakov et al. 2015). Therefore, IL-22BP is extensively used as an effective competitive inhibitor of IL-22 signaling in vitro (Mühl and Bachmann 2019; Trevejo-Nunez et al. 2019; Fukaya et al. 2018).

After IL-22 forms a complex with IL-22R1 and IL-10R2, STAT3 phosphorylation is induced by activated Janus kinase (JAK) signaling molecules tyrosine kinase 2 (IL-10R2 activator) and JAK1 (IL-22R1 activator) (Sabat et al. 2014; Ziesché et al. 2007). In primary cells exposed to IL-22, STAT3 at residues Ser727 and Tyr705 is phosphorylated (Sestito et al. 2011; Xue et al. 2017). Besides STAT3, phosphorylation of STAT5 and STAT1 has also been reported (Lejeune et al. 2002; Wolk et al. 2004). Interestingly, STAT3 acetylation at lysine 686 is essential for the phosphorylation of Tyr705, and the deacetylase sirtuin 1 suppresses IL‑22‑induced activation of STAT3 and cellular effects (Sestito et al. 2011; Yuan et al. 2005; Nie et al. 2009). These modifications of STATs promote their molecular dimerization, allowing them to translocate to the nucleus and regulate the expression of their target genes. It has been reported that STAT molecules including STAT3, STAT1, and STAT5 play key roles in lipid metabolism and inflammatory response (Piaszyk-Borychowska et al. 2019; Chen et al. 2019a; Shi et al. 2020b). Recently, Chen et al. summarized the crucial roles of STAT3 in macrophage polarization, endothelial cell dysfunction, immunity, and inflammation during atherosclerosis (Chen et al. 2019a). Thus, targeted inhibition of STAT signaling molecules may be an important potential therapeutic strategy for atherosclerosis. In addition, IL-22 also utilizes Tyk2 and Jak1 to transmit other downstream phosphorylation signals, such as protein kinase B (PKB, also called Akt), mitogen-activated protein kinase (MAPK), P38, c-Jun N-terminal kinase (JNK), and extracellular signal-regulated kinase-1/2 (ERK1/2) (Lejeune et al. 2002; Wolk et al. 2004; Wolk et al. 2010; Andoh et al. 2005; Pickert et al. 2009). Of note, these signaling molecules are also closely associated with the progression of atherosclerosis (Linton et al. 2019; Reustle and Torzewski 2018; Chen et al. 2015).

## Role of IL-22 in atherosclerosis

Atherosclerosis is known as a chronic and progressive pathologic process underlying cardiovascular disease. The precise role of IL-22 in atherosclerosis is still controversial, although most studies suggest a proatherogenic function of IL-22. apoE/IL-22 double knockout mice exhibit decreased plaque size both in the aortic root and the aorta compared with apoE knockout controls (Rattik et al. 2015). Moreover, in another study of atherosclerosis, IL-22R1 and IL-22 are expressed in mouse atherosclerotic plaques, and their expression levels are significantly elevated in apoE knockout mice (Shi et al. 2020a). Furthermore, the authors also showed that treatment with recombinant mouse IL-22 (rIL-22) markedly aggravates atherosclerosis development in apoE^−/−^ mice fed a high-fat diet (HFD), whereas blocking circulating IL-22 with a neutralizer of IL-22 (IL-22 mAb) substantially reduces the atherosclerotic lesion area (Shi et al. 2020a). S100/calgranulin, a pro-inflammatory protein, promotes the development of atherosclerosis with excessive cholesterol accumulation in atherosclerotic lesions in apoE-null mice (Chellan et al. 2014). Further study revealed that myeloid-derived S100/calgranulin upregulates IL-22 in a receptor for advanced glycation endproducts (RAGE)-dependent manner, resulting in impaired cholesterol homeostasis in macrophages (Chellan et al. 2014). Besides, circulating IL-22 levels are markedly higher in peripheral blood of patients with unstable angina and acute myocardial infarction (AMI) compared to stable angina and healthy control subjects (Zhang et al. 2013; Xia et al. 2012). These studies suggest that IL-22 contributes to plaque growth and promotes plaque instability during lesion formation. However, in the LDLR^−/−^ mouse model, IL-22 plays a protective role in atherosclerosis (Fatkhullina et al. 2018). Inactivation of IL-22 exacerbates atherosclerosis in LDLR^−/−^ mice exposed to the western diet (WD) by increasing pro-atherogenic metabolites such as trimethylamine N-oxide (TMAO) and lipopolysaccharide (LPS) (Fatkhullina et al. 2018). The contradictory results may be due to the different experimental conditions, including diets and mouse models.

## IL-22 promotes atherosclerosis through multiple mechanisms

**Fig. 1:**
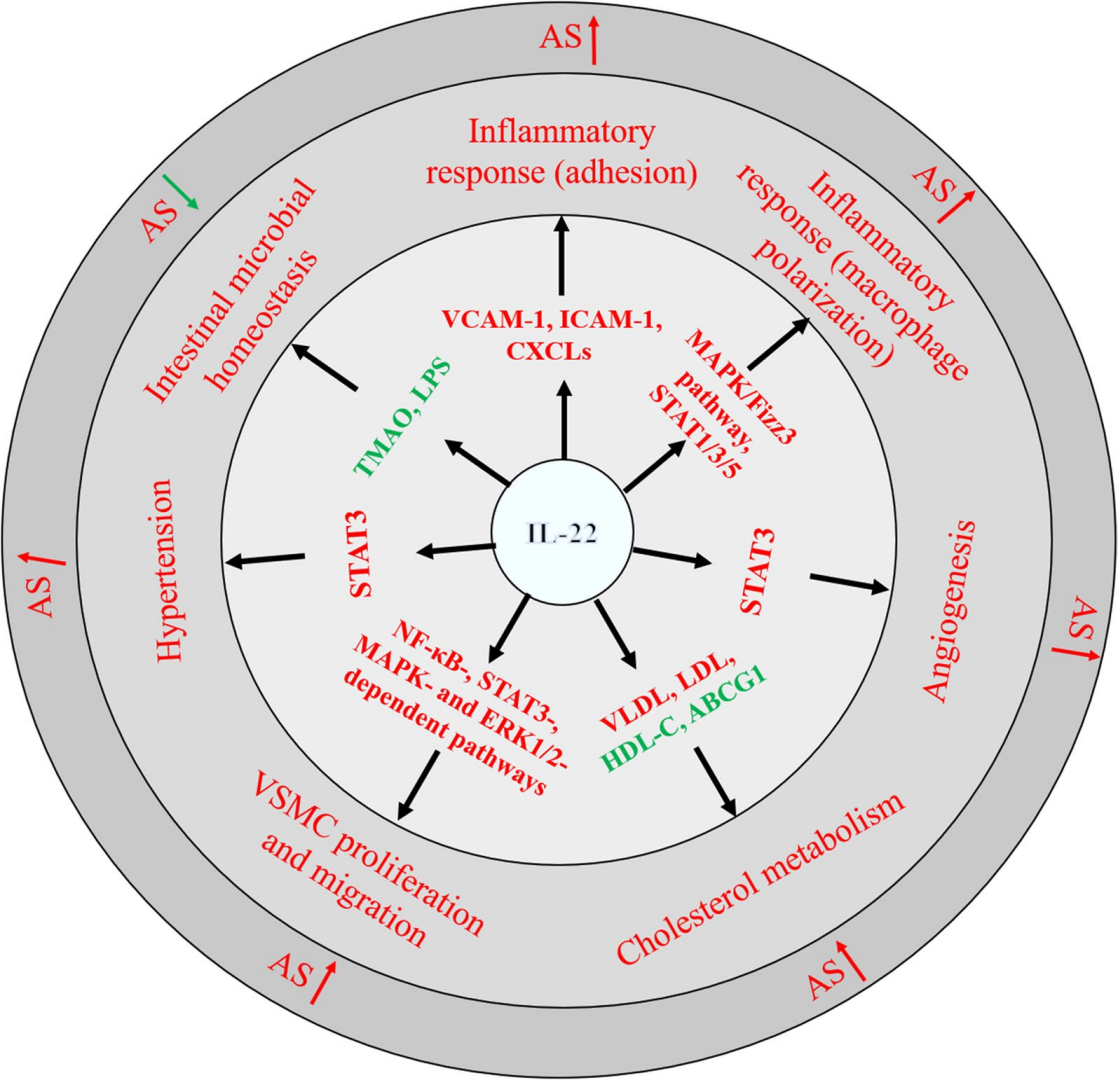
IL-22-mediated regulation and the major cardiometabolic risk factors of atherosclerosis. *VCAM-1* vascular cell adhesion molecule-1, *ICAM-1* Intercellular adhesion molecule-1, *CXCLs* CXC chemokine ligands, *MAPK* mitogen-activated protein kinase, *STAT* signal transducer and activator of transcription, *VLDL* very low-density lipoprotein, *LDL* low-density lipoprotein, *HDL-C* high-density lipoprotein cholesterol, *ABCG1* ATP-binding cassette transporter G1, *NF-κB* nuclear transcription factor-κB, *ERK* extracellular signal-regulated kinase, *VSMC* vascular smooth muscle cell, *TMAO* trimethylamine N-oxide, *LPS* lipopolysaccharide, *AS* atherosclerosis. Arrows in red: promote; Arrows in green: inhibit; text in red: proatherogenic changes; text in green: antiatherogenic changes

The pathophysiology of atherosclerosis is a multifactorial and complex process involving inflammation, angiogenesis, dysregulation of cholesterol metabolism, VSMC proliferation and migration, and hypertension (Chistiakov et al. 2015; Jaipersad et al. 2014; Kostov and Halacheva 2018). As a unique and pleiotropic cytokine, IL-22 promotes the development of atherosclerosis through multiple mechanisms (Fig. 1).

### IL-22 in inflammatory response

Atherosclerosis is considered to be a progressive chronic inflammatory disease of the vascular wall. It is known that T cells can trigger the inflammatory response in the vascular wall by secreting numerous pro-inflammatory mediators (Libby and Hansson 2018). In particular, cytokines are important contributors to complex inflammatory reactions in the vascular wall and atherosclerotic plaques.

### IL-22 regulates adhesion molecules

Adhesion molecules play a key role in atherogenesis by promoting the adhesion of monocytes and leukocytes to activated endothelial cells and inducing subsequent vascular inflammation (Galkina and Ley 2007). Intercellular adhesion molecule-1 (ICAM-1) and vascular cell adhesion molecule-1 (VCAM-1) are considered important risk factors involved in the development of atherosclerosis. Upon IL-22 interacts with its receptor, it can induce cells to produce a variety of adhesion molecules and chemokines to participate in the inflammatory response. It has been reported that IL-22 induces cells to produce ICAM-1 and VCAM-1 (Hoeven et al. 2018). In addition to ICAM-1 and VCAM-1, CXC chemokine ligands (CXCL) have also been identified as key adhesion molecules in adhesion, migration, and recruitment of monocytes during the pathogenesis of atherosclerosis. Some clinical data have demonstrated that IL-22 levels are strikingly elevated in patients with atherosclerosis cardiovascular disease, which positively correlated with the proinflammatory cytokine levels (CXCL-11, CXCL-10, and CXCL-9) in carotid plaques (Xia et al. 2012). Moreover, as a pro-inflammatory cytokine, IL-22 was also reported to synergy with TNF-α in promoting the induction of CXCL1, CCL2, CCL5, CXCL5, CCL20, and CCL26 expression (Aujla and Kolls 2009). In addition, STAT1 and STAT5 were reported to act as a proinflammatory signal in the regulation of expression of adhesion molecules and chemokines (Coccia et al. 1999; Lian et al. 2016; Soriano et al. 2003; Yang et al. 2007). Given that IL-22 is a critical regulator of STAT signaling molecules. STAT1 and STAT5 may be important mechanisms involved in the expression of adhesion molecules regulated by IL-22.

### IL-22 regulates macrophage phenotypes

It is well known that tissue-resident and monocyte-derived recruited macrophages are pivotal regulators of tissue repair, regeneration, and fibrosis. Macrophages can be functionally and phenotypically modified to adopt pro-wound-healing, pro-resolving, pro-inflammatory, tissue-regenerating, anti-inflammatory, anti-fibrotic, and pro-fibrotic properties in response to microenvironmental stimuli (Wynn and Vannella 2016). Of note, pro-inflammatory macrophages are primarily associated with inflammatory activity and have proatherogenic properties, whereas anti-inflammatory macrophages are mainly responsible for inhibiting inflammatory responses and increasing atherosclerotic plaque stability (Moore and Tabas 2011; Tabas and Bornfeldt 2016; Li et al. 2018). Several lines of evidence suggest that IL-22 is closely associated with pro-inflammatory macrophage phenotype (Hou et al. 2018; Yang et al. 2019). Deletion of IL-22 was reported to markedly induce cardiac macrophage toward an anti-inflammatory phenotype (Ye et al. 2020). The underlying mechanism involves the mitogen-activated protein kinase (MAPK)/Fizz3 pathway. Moreover, IL-22 levels increase significantly during the differentiation of bone marrow-derived macrophages into pro-inflammatory phenotype under the stimulation of lipopolysaccharide (LPS) (Yang et al. 2019). The toll-like receptor 4 (TLR4) signaling pathway plays an important role in the regulation of macrophage inflammation. It has been reported that TLR4 deficiency induces the differentiation of alveolar macrophages toward an anti-inflammatory phenotype, accompanied by a decrease in IL-22 expression (Shen and Liu 2015). Besides, STAT molecules such as STAT1 and STAT5 were found to drive macrophage differentiation toward a pro-inflammatory phenotype (Gan, et al. 2017; Lawrence and Natoli 2011). STAT1 and STAT5 are also known to trigger inflammatory cascade (e.g., NF-κB, TLR4, MAPK) that promotes macrophage inflammatory response and atherogenesis (Chmielewski et al. 2016; Wu et al. 2020; Shen-Orr et al. 2016). In contrast, inhibition of STAT1 and STAT5 attenuates inflammatory response in atherosclerosis (Sikorski et al. 2011a, b; Wang et al. 2021). Of note, IL22R1 is widely expressed in macrophages. The IL-22/IL22R1 complex is a key activator of STAT signaling molecules. Thus, the IL-22/IL22R1 complex may direct macrophages towards a pro-inflammatory phenotype via regulating STAT1 and STAT5.

### IL-22 in angiogenesis

Angiogenesis, defined as the formation of new microvessels (small venules and capillaries) from pre‐existing blood vasculature, plays a crucial role in plaque growth and instability (Camaré et al. 2017). IL-22 and its receptor IL-22R1 were significantly upregulated in endothelial cells following ischemic injury (Hu et al. 2018). In vivo studies further showed that IL-22 promotes the formation of new blood vessels in a mouse hindlimb ischemia model. Also, IL-22 directly acts on human umbilical vein endothelial cells (HUVECs) and induces HUVEC proliferation, migration, and tube formation in vitro (Hu et al. 2018). Blocking IL-22 with IL-22 mAb reversed the above effects. In addition to regulating angiogenesis in peripheral arterial disease, IL-22 was also found to promote neovascularization in a mouse tumor model (Protopsaltis et al. 2019; Shang et al. 2015). Importantly, IL-22 treatment strikingly increased phosphorylation of STAT3 in HUVECs (Hu et al. 2018; Shang et al. 2015). In contrast, treatment with STAT3 inhibitor or knockdown of IL-22R1 attenuated IL-22-induced endothelial cell survival or tube formation (Hu et al. 2018; Shang et al. 2015). These studies indicate that STAT3 is an important signaling molecule required for IL-22/IL22R1 complex-induced angiogenesis. However, the key proteins involved in IL-22-induced angiogenesis still need to be further confirmed.

### IL-22 in cholesterol metabolism

Lipid metabolic disorder, especially hypercholesterolemia, is an important risk factor for atherosclerosis. High-density lipoprotein cholesterol (HDL-C) exerts an atheroprotective effect, but very low-density lipoprotein (VLDL) and low-density lipoprotein cholesterol (LDL-C) are proatherogenic. It has been reported that circulating IL-22 is negatively associated with HDL-C levels, while it was positively associated with LDL and VLDL levels (Torquati et al. 2019; Lodge et al. 2021). Thus, IL-22 may contribute to the development of hypercholesterolemia.

The accumulation of foam cells (cholesterol-laden macrophages) in the arterial walls plays an important role in the pathogenesis of atherosclerosis. ATP-binding cassette transporter A1 (ABCA1) and ABCG1, two important transmembrane transporters, are widely expressed by macrophages and play a pivotal role in promoting the efflux of cholesterol onto apoA-I and HDL (Tarling and Edwards 2011; Ren et al. 2019). CD36, lectin-like oxidized low-density lipoprotein receptor-1 (LOX-1), and scavenger receptor class A (SRA) are primarily responsible for intracellular cholesterol uptake (Crucet et al. 2013). A recent study showed that recombinant IL-22 decreases ABCG1 expression and impairs macrophage cholesterol efflux (Chellan et al. 2014). However, IL-22 does not affect the expression of LOX-1, CD36, and ABCA1. Of note, the activity of ABCG1 in macrophages is largely modulated by the nuclear transcription factor liver X receptor (LXR) (Larrede et al. 2009). Interestingly, IL-22 does not decrease cholesterol efflux in macrophages pretreated with TO-901317 (an LXR agonist) (Chellan et al. 2014), suggesting that endogenous upregulation of ABCG1 diminishes the harmful effect of IL-22 on macrophage cholesterol efflux. In addition, Agrawal et al. found that STAT1 is essential for optimal foam cell formation and the development of atherosclerotic lesions (Agrawal et al. 2007). Inhibition of STAT1 in macrophages significantly suppresses oxidized low-density lipoprotein (oxLDL)-induced foam cell formation (Agrawal et al. 2007). In addition to STAT1, STAT5 has also been reported to participate in foam cell formation. STAT5 inhibitor markedly reduces cholesterol accumulation in oxLDL-stimulated macrophages (Wang et al. 2021). Therefore, it is possible that IL-22 promotes cholesterol accumulation via the regulation of STAT1 and STAT5.

### IL-22 in VSMC proliferation and migration

It is well known that VSMC proliferation and migration from the media to the intima are pivotal cellular events in the progression of atherosclerosis and the VSMC phenotypic regulation (transformation from contractile type to synthetic type) plays a crucial role in this process (Basatemur et al. 2019). Rattik et al. found that IL-22R1 is expressed by VSMCs in the mouse vascular wall and deletion of IL-22 leads to the development of less collagen-rich and smaller plaques in apoE^−/−^ mice (Rattik et al. 2015). Furthermore, increased expression of genes related to VSMC contraction (vinculin, calmodulin, α-actin) was observed in arteries from HFD fed apoE/IL-22 double-knockout mice (Rattik et al. 2015). However, there is no difference in the expression of markers related to VSMC migration or synthesis phenotype such as vimentin, platelet-derived growth factor (PDGF), smoothelin, or matrix metalloproteinase 9 (MMP9) (Rattik et al. 2015). Thus, IL-22 mainly affects the early stage in the VSMC phenotypic switch but not VSMC functional features such as migration. In contrast, apoE^−/−^ mice treated with recombinant mouse IL-22 showed reduced VSMC α-actin expression and increased collagen contents (Shi et al. 2020a). Several studies demonstrated that IL-22 enhances the proliferation and migration of SMCs through NF-κB-, STAT3-, MAPK- and ERK1/2-dependent pathways (Bansal et al. 2013; Chang et al. 2011). Collectively, IL-22 acts as an important cytokine in the regulation of VSMC proliferation and migration and may be a promising therapeutic target for atherosclerosis.

### IL-22 in hypertension

Hypertension is known as an important risk factor for the development of atherosclerosis. Recent studies showed that IL-22 has a close relationship with human hypertension, and IL-22 is regarded as a promising therapeutic target for hypertension (Ye et al. 2017; Zhong et al. 2020). The plasma levels of IL-22 were markedly higher in patients with hypertension than in health participants, and IL-22 levels were strikingly elevated and positively correlated with diastolic blood pressure and systolic blood pressure (Ye et al. 2017). Moreover, after treatment with recombinant mouse IL-22 in mice injected with angiotensin II, the interaction of IL-22 with IL-10R2 and IL-22R1 can activate the STAT3 signaling pathway, aggravating angiotensin II-induced inflammatory responses, endothelial dysfunction, and hypertension (Ye et al. 2017). Conversely, treatment with the anti-IL-22 neutralizing MAbs has an opposite biological function (Ye et al. 2017).

## Positive regulators of IL-22 in atherosclerosis

### IL-23

IL-23 plays a pro-inflammatory role in atherosclerosis. IL-23 aggravates atherosclerosis by increasing the release of inflammatory cytokines and the production of reactive oxygen species (ROS) in macrophages (Abbas et al. 2015; Döring 2015). Also, IL-23 is a crucial marker of pro-inflammatory macrophages. IL-23 is one of the major inducers of IL-22 production and expression (Kastelein et al. 2007). IL-23 promotes IL-22 expression via affecting a variety of cells including macrophages. It has been reported that IL-23 increases the expression of IL-22, which results in enhanced IL-23R expression (Sano et al. 2015). This leads to enhanced interaction between IL-23 and its receptor, and therefore promotes the production of IL-22. Also, IL-23 promotes the phosphorylation of phosphatidylinositol-3-kinase (PI3K)/Akt and inhibitor κB-α (IκB-α), which activate the transcription factors STAT3- and NF-κB-dependent signaling in target cells, respectively (Cho et al. 2006). Thus, IL-23 may be an important regulator that synergistically enhances the pro-atherosclerotic role of IL-22.

### IL-7

IL-7, a key cytokine for the proliferation and development of T cells, drives inflammation in atherogenesis and promotes plaque instability in coronary artery disease involving interactions between chemokines, platelets, and monocytes (Damås et al. 2003; Arya et al. 2012). IL-7 can positively modulate the expression and production of IL-22 (Cella et al. 2010). However, IL-7 is unlikely to directly regulate IL-22 expression. IL-7 enhances the expression of retinoid-related orphan receptor gamma-t (RORγt), which is an important transcription factor for IL-22 expression (Vonarbourg et al. 2010). Furthermore, blockade of IL-7R signaling or IL-7Rα deficiency reduces RORγt expression in IL-22-producing cells (Vonarbourg et al. 2010). RORγt was reported to augment IL-22 enhancer activity by directly binding to the conserved noncoding sequence 32 (CNS 32) (Sekimata et al. 2019). Also, RORγt regulates the differentiation of IL-22-producing cells to achieve the optimal conditions for IL-22 expression (Qiu et al. 2012; Nurieva et al. 2007; Sanos et al. 2009). Taken together, the IL-7/IL-7R pathway plays an important role in the regulation of IL-22 production by enhancing RORγt expression.

### Aryl hydrocarbon receptor

The aryl hydrocarbon receptor (AhR), an important ligand‑activated transcription factor, has been reported to accelerate atherosclerosis through triggering vascular inflammation and promoting foam cell formation (Dahlem et al. 2020; Wu et al. 2011). AhR can directly modulate the expression and production of IL-22 gene or regulate the development and production of Th17 and innate lymphoid cells (ILCs) (IL-22-producing cells) (Qiu et al. 2012; Veldhoen et al. 2008). AhR is typically located in the cytoplasm in a complex with Hsp90 chaperones until the binding of its ligand induces a conformational change, leading to the separation of AhR from heat shock protein 90 (HSP90) (Esser et al. 2009). Then, AhR enters the nucleus together with its ligand and directly increases the expression of IL-22 gene. Of note, this transcriptional regulation does not depend on RORγt but may require the generation of endogenous AhR ligands by the Notch signaling pathway (Alam et al. 2010). Potential endogenous AhR ligands could be derived from the gut microbiota, or from the diet (Lee et al. 2011), including 2,3,7,8-tetrachlorodibenzo-p-dioxin (TCDD) and indol-3-carbinol, 6-formylindolo[3,2-b] carbazole (FICZ) (Nguyen and Bradfield 2008; Denison and Nagy 2003). It has been reported that FICZ is involved in Notch-mediated IL-22 expression and production (Oberg et al. 2005). In addition, several lines of evidence indicate that AhR also acts independently of IL-23 (Veldhoen et al. 2008, 2009). However, it is unclear whether AhR can act downstream or upstream of the IL-23/IL-23R pathway.

### IL-1β

IL-1β, a classic proinflammatory cytokine, has been shown to promote atherosclerotic plaque progression through increasing activation and proliferation of VSMCs and enhancing the synthesis of adhesion molecules on endothelial cells (Khan et al. 2018; Tumurkhuu et al. 2018). Also, high IL-1β concentrations are associated with an increased risk of hypertension (Krishnan et al. 2014; Alfaidi et al. 2018). Similar to IL-22, IL-1β is expressed in a variety of cell types related to the development of atherosclerosis including endothelial cells, macrophages, and VSMCs (Sims and Smith 2010). Interestingly, IL-1β was found to promote IL-22 production independently of IL-23, although they can synergistically induce the production of IL-22 (Sutton et al. 2009). Notably, constant IL-1β signaling is essential for sustained expression of AHR and IL-22, which is different from IL-23 (Hughes et al. 2010). Moreover, ablation of IL-1R1 signaling diminishes IL-22 expression and production (Hughes et al. 2010). Taken together, these data suggest that the IL-1β/ IL-1R1/IL-22 axis may play a key role in the aetiology of atherosclerosis.

### Notch

The Notch signaling pathways were found to promote atherosclerosis by regulating pro-inflammatory macrophage differentiation, endothelial dysfunction, and angiogenesis (Fukuda et al. 2012; Mao and Jiang 2018; Pitulescu et al. 2017). The Notch signaling is essential for the specific differentiation and development of IL-22-producing cells (Rankin et al. 2013; Mielke et al. 2013). The Notch can stimulate CD4^+^ T cells to increase IL-22 production (Murano et al. 2014). Moreover, the Notch enhances IL-22-induced STAT3 expression through its target gene Hes1, thereby affecting IL-22 targets and IL-22 production (Murano et al. 2014). In contrast, IL-22 signaling and production are abrogated in Notch-deficient mice (Alam et al. 2010; Murano et al. 2014). In addition, it has been found that the Notch signaling promotes the expression and production of IL-22 through upregulating transcription factors AhR (Lee et al. 2011; Weidenbusch et al. 2017) and recombination signal binding protein‐Jκ (RBP‐J) (Alam et al. 2010). These studies suggest that multiple transcription factors are involved in Notch signaling-mediated IL-22 expression.

## Negative regulators of IL-22 in atherosclerosis

### TGF-β

Transforming growth factor‐β (TGF‐β) signaling plays a crucial role in the development and progression of atherosclerosis. Many studies demonstrated that activation of TGF‐β signaling suppresses the development of atherosclerosis via regulating a variety of processes, including angiogenesis, VSMC proliferation and migration, inflammation, and foam cell formation (Grainger 2007; Chen et al. 2019b). TGF-β signaling was reported to serve as a suppressor of IL-22 expression and production in a dose-dependent manner (Rutz et al. 2011; Volpe et al. 2009; Penel-Sotirakis et al. 2012). However, IL-23 can override the effect of TGF-β on IL-22, thus promoting IL-22 expression and production (Volpe et al. 2009; Zhou et al. 2008). c-musculoaponeurotic fibrosarcoma (c-Maf) is a basic region leucine zipper (bZIP)-type transcription factor, which belongs to the Maf family. It has been reported that c-Maf specifically binds to the promoter of IL-22 gene and serves as a transcriptional repressor for IL-22 (Rutz et al. 2011). Moreover, in human T and invariant natural killer T (iNKT) cells, TGF-β signaling was found to suppress IL-22 expression by activating c-Maf (Rutz et al. 2011). Of note, TGF-β signaling does not suppress the production of IL-22 through the inhibition of AhR, BATF, or RORγt (Rutz et al. 2011). Thus, c-Maf is an important transcription factor for TGF-β signaling-mediated IL-22 expression in T cells. It is necessary to determine whether c-Maf functions in a similar manner in other cell types for which TGF-β was shown to inhibit IL-22.

### IL-27

IL-27 was reported to prevent atherosclerosis in LDLR^−/−^ mice by inhibiting the activation of macrophages (Hirase et al. 2013). Furthermore, knockout of IL-27 or IL-27 receptor in bone marrow-derived macrophages aggravates atherosclerosis and facilitates the activation of bone marrow-derived cells in the arterial wall (Hirase et al. 2013; Koltsova et al. 2012). IL-27 has also been identified as a negative modulator of IL-22 expression. IL-27 was found to inhibit the expression and production of IL-22 by upregulating suppressor of cytokine signaling 1 (SOCS1) expression (Wang et al. 2013). SOCS proteins are known as negative regulators of the Jak/STAT signal transduction (Yoshimura et al. 2007). AG490, an inhibitor of Jak2/STAT signaling, significantly blocks the inhibition of IL-22 production mediated by IL-27 (Wang et al. 2013). Besides, IL-27 also induces c-Maf expression, which inhibits the expression of IL-22 (Pot et al. 2009). Thus, IL-27 may attenuate the pro-atherosclerotic role of IL-22 by upregulating c-Maf and SOCS1 expression.

### IL-38

IL-38 is known as an anti-inflammatory factor that suppresses pro-inflammatory cytokine production by its receptors and protects against cardiovascular disease (Graaf et al. 2021; Esmaeilzadeh et al. 2019). Recently, IL-38 overexpression was found to inhibit hyperlipidemia and atherosclerosis in apoE^−/−^ by alleviating inflammation (Yang et al. 2018). IL-38 can regulate IL-22 expression in a dose-dependent manner. At low concentrations, IL-38 suppresses IL-22 production, whereas, at high concentrations, IL-38 modestly increases IL-22 production (Veerdonk et al. 2012; Tortola et al. 2012). Thus, further studies are needed to explore the exact mechanism by which IL-38 regulates IL-22 expression and production in atherosclerosis.

### IL-25

IL-25 was reported to inhibit the development of atherosclerosis by decreasing inflammation and oxidized LDL-specific antibodies (Mantani et al. 2015; Mantani et al. 2018a). Also, IL-25 appears to be expressed by cells commonly found in atherosclerotic plaques such as endothelial cells, VSMCs, and macrophages (Mantani et al. 2018a, b). It has been reported that serum levels of IL-25 are significantly correlated with IL-22 in rheumatoid arthritis patients (Min et al. 2020). Addition of IL-25 markedly reduces IL-22 expression but not changes mRNA levels of IL-22 receptors, IL-10RB and IL-22R1 (Min et al. 2020), suggesting that the suppressive role of IL-25 on IL-22 expression is independent with IL-22 receptors. Further studies showed that IL-25 also inhibits IL-22-induced STAT3 and p38 MAPK/IκBα pathway (Min et al. 2020). Thus, upregulation of IL-25 may alleviate atherosclerosis by reducing IL-22 expression.

## Conclusions and future directions

IL-22, an important member of the IL-10 cytokine family, has attracted growing interest in recent years. IL-22 plays a critical role in the development of atherosclerosis through its involvement in the modulation of angiogenesis, inflammatory response, hypertension, VSMC proliferation and migration, and cholesterol metabolism (Fig. 1). Also, IL-22 may be regulated by other cytokines and proteins during atherosclerosis (Table 1). However, the immunoregulatory role of IL-22 appears complicated. Although numerous data have established the proatherogenic effects, a recent study revealed a paradoxical result that IL-22 protects against WD-induced atherosclerosis in LDLR^−/−^ mice by regulating intestinal microbial homeostasis (Fatkhullina et al. 2018). The paradoxical effects of IL-22 on immunoregulation may be diet- or mouse model-specific effects, which should be carefully considered when we use drugs to modulate IL-22 expression and production in vivo. In addition, it has been reported that IL-22R1 is expressed in mouse atherosclerotic plaques, and its expression levels are increased in ApoE^−/−^ mice (Shi et al. 2020a). However, the expression of other IL-22 receptors (IL-10R and IL-22BP) in atherosclerosis remains largely unknown. Overall, increasing studies support the view that targeting IL-22 may be a promising therapeutic strategy for atherosclerosis.

**Table 1:** Potential regulators of IL-22 expression and production in atherosclerosis

Molecules	Type of regulation	Transcription factor	Pathway	Potential role in AS	References
IL-23	Positive	STAT3, NF-κB	IL-23/IL-23R, PI3K/Akt, IκB-α	Promotes inflammatory responses	Sano et al. 2015; Cho et al. 2006)
IL-7	Positive	RORγt	IL-7/IL-7R	Promotes inflammatory responses	Vonarbourg et al. 2010; Sekimata et al. 2019)
AhR	Positive	–	Notch/FICZ	Promotes inflammatory responses and cholesterol accumulation	Qiu et al. 2012; Veldhoen et al. 2008)
IL-1β	Positive	AHR	IL-1β/IL-1R1	Promotes inflammatory responses and VSMC proliferation and migration	Sutton et al. 2009; Hughes et al. 2010)
Notch	Positive	AhR, RBP‐J, STAT3	Notch-Hes1	Promotes inflammatory responses, endothelial dysfunction, and angiogenesis	Rankin et al. 2013; Mielke et al. 2013; Murano et al. 2014)
TGF-β	Negative	c-Maf	TGF-β signaling	Suppresses angiogenesis, VSMC proliferation and migration, inflammation, and cholesterol accumulation	Rutz et al. 2011; Volpe et al. 2009; Penel-Sotirakis et al. 2012)
IL-27	Negative	SOCS1, c-Maf	Jak/STAT signaling	Suppresses inflammatory responses	Wang et al. 2013; Pot et al. 2009)
IL-38	Negative	–	IL-38- IL-36R	Suppresses inflammatory responses	Veerdonk et al. 2012; Tortola et al. 2012)
IL-25	Negative	STAT3	p38 MAPK/IκBα	Suppresses inflammatory responses	Min et al. 2020)

– Not determined, *AS* atherosclerosis

Numerous studies demonstrated that VSMCs, endothelial cells, and macrophages are the major effector cells involving atherosclerosis (Cho et al. 2011; Menon and Fisher 2015; Raman and Khanal 2021). Importantly, IL-22 and IL-22R1 are widely expressed in these cells. Also, increasing evidence suggests that IL-22 may promote atherosclerosis through multiple mechanisms by these effector cells. Thus, these cells may be the main target for IL-22 in atherosclerosis. In the future, more data are required to reveal the exact role of IL-22 in these effector cells during the development of atherosclerosis. Although many studies have demonstrated the beneficial effects of decreased expression, it is currently difficult to speculate on the most effective method to inhibit IL-22 expression in atherosclerosis. Targeting IL-22 or its receptor complex is the most direct method to inhibit IL-22-mediated functions. Preclinical and clinical studies have demonstrated that a human anti-IL-22-mAb (fezakinumab) and IL‑22R1‑targeting mAbs are currently tested as a treatment for several inflammatory diseases such as rheumatoid arthritis and severe atopic dermatitis, without obvious adverse safety concerns observed (Sabat et al. 2014; Kragstrup et al. 2018; Guttman-Yassky et al. 2018). Atherosclerosis is also known as an inflammatory disease; however, it is not clear whether these therapeutic strategies can protect against the development of atherosclerosis. Therefore, both clinical and basic studies are needed to validate this possibility. Besides, there are several crucial and insightful questions that require to be answered. First, both innate and adaptive immunity play important roles in the development of atherosclerosis (Hansson et al. 2002; Miteva et al. 2018). The crosstalk of IL-22 and innate and adaptive immunity in atherosclerosis is worthy of attention. In particular, monocytes/macrophages are known as key immune cells, which have important proatherogenic effects. Th1 response has a potent proatherogenic effect, but regulatory T cells and some Th2-related cytokines play anti-atherosclerotic protective roles. Thus, it is necessary to explore which immune cells-derived IL-22 affects these atherosclerosis-related immune cells. Second, how to most effectively target IL‑22, either by transcriptional or posttranscriptional modulation? Third, as an important proinflammatory cytokine, does IL‑22 play a role in pyroptosis (a pivotal driver of atherosclerosis)? Fourth, do a specific and efficient method to counteract the detrimental actions of IL‑22 on atherogenesis exist in vivo? Finally, reverse cholesterol transport (RCT), an important protective mechanism against the development of atherogenesis, is a dynamic process in which excessive cholesterol from extrahepatic tissues is delivered to the liver for further metabolism and biliary excretion (Rader et al. 2009; Li et al. 2021). Therefore, it is of importance to detect whether IL‑22 can increase the efficiency of RCT. The answers to these sorts of questions will undoubtedly provide unique insights into the roles of IL‑22 in atherosclerosis and make IL‑22 an attractive therapeutic target for therapy aiming at the reduction of atherogenesis.

## Data Availability

Not applicable.

## Abbreviations

CVDs: Cardiovascular diseases
JAK: Janus kinase
VSMC: Vascular smooth muscle cell
IL-22: Interleukin-22
MMP9: Matrix metalloproteinase 9
VLDL: Very low-density lipoprotein
MAbs: Monoclonal antibodies
STAT: Signal transducer and activator of transcription
HFD: High-fat diet
GSK3β: Glycogen synthase kinase 3β
IL-22BP: IL-22 binding protein
VCAM-1: Vascular cell adhesion molecule-1
WD: Western diet
JNK: C-Jun N-terminal kinase
TLR4: The toll-like receptor 4
rIL-22: Recombinant mouse IL-22
SRA: Scavenger receptor class A
RAGE: Receptor for advanced glycation endproducts
MAPK: Mitogen-activated protein kinase
AMI: Acute myocardial infarction
TMAO: Trimethylamine N-oxide
LPS: Lipopolysaccharide
LOX-1: Lectin-like oxidized low-density lipoprotein receptor-1
ICAM-1: Intercellular adhesion molecule-1
bZIP: Basic region leucine zipper
HUVECs: Human umbilical vein endothelial cells
HDL-C: High-density lipoprotein cholesterol
CXCL: CXC chemokine ligands
ERK1/2: Extracellular signal-regulated kinase-1/2
PDGF: Platelet-derived growth factor
LXR: Liver X receptor
AhR: Aryl hydrocarbon receptor
ILCs: Innate lymphoid cells
TGF‐β: Transforming growth factor‐β
LDL-C: Low-density lipoprotein cholesterol
c-Maf: C-musculoaponeurotic fibrosarcoma
ABCA1: ATP-binding cassette transporter A1
iNKT: Invariant natural killer T
SOCS1: Suppressor of cytokine signaling 1
RCT: Reverse cholesterol transport
WHO: World Health Organization
